# Resting-state EEG and MEG biomarkers of pathological fatigue – A transdiagnostic systematic review

**DOI:** 10.1016/j.nicl.2023.103500

**Published:** 2023-08-18

**Authors:** Henrik Heitmann, Paul Theo Zebhauser, Vanessa D. Hohn, Peter Henningsen, Markus Ploner

**Affiliations:** a Department of Neurology, School of Medicine, Technical University of Munich (TUM), Germany; b TUM-Neuroimaging Center, School of Medicine, Technical University of Munich (TUM), Germany; c Department of Psychosomatic Medicine and Psychotherapy, School of Medicine, Technical University of Munich (TUM), Germany

**Keywords:** EEG, MEG, Fatigue, Transdiagnostic, Biomarker, Systematic review

## Abstract

Fatigue is a highly prevalent and disabling symptom of many disorders and syndromes, resulting from different pathomechanisms. However, whether and how different mechanisms converge and result in similar symptomatology is only partially understood, and transdiagnostic biomarkers that could further the diagnosis and treatment of fatigue are lacking. We, therefore, performed a transdiagnostic systematic review (PROSPERO: CRD42022330113) of quantitative resting-state electroencephalography (EEG) and magnetoencephalography (MEG) studies in adult patients suffering from pathological fatigue in different disorders. Studies investigating fatigue in healthy participants were excluded. The risk of bias was assessed using a modified Newcastle-Ottawa Scale. Semi-quantitative data synthesis was conducted using modified albatross plots. After searching MEDLINE, Web of Science Core Collection, and EMBASE, 26 studies were included. Cross-sectional studies revealed increased brain activity at theta frequencies and decreased activity at alpha frequencies as potential diagnostic biomarkers. However, the risk of bias was high in many studies and domains. Together, this transdiagnostic systematic review synthesizes evidence on how resting-state M/EEG might serve as a diagnostic biomarker of pathological fatigue. Beyond, this review might help to guide future M/EEG studies on the development of fatigue biomarkers.

## Highlights

• Systematic review of potential transdiagnostic EEG-based biomarkers of fatigue.
• Increased theta and decreased alpha power in patients with fatigue.
• Potential transdiagnostic biomarker and future treatment target in fatigue.

## 1 Introduction

Fatigue, i.e., the feeling of overwhelming mental or physical tiredness and exhaustion, is a frequent symptom of many disorders. It is highly prevalent in neuropsychiatric disorders (Penner and Paul, 2017), inflammatory-rheumatic diseases (Davies et al., 2021), cancer (Bower, 2014), and post-infectious syndromes including long COVID (Campos et al., 2022, Davis et al., 2023). Moreover, it is a core feature of Myalgic Encephalomyelitis/Chronic Fatigue Syndrome (CFS) (Marshall-Gradisnik and Eaton-Fitch, 2022) and Fibromyalgia Syndrome (FMS) (Salaffi et al., 2020). In these disorders, fatigue is highly disabling, strongly interferes with quality of life, and is a significant cause of early retirement (Penner and Paul, 2017, Davies et al., 2021). Treatment of this burdensome symptom is often unsatisfactory (Davies et al., 2021). Pharmacological treatments mainly comprise psychostimulants, and non-pharmacological treatments include cognitive-behavioral approaches, relaxation techniques, and graded exercise, with modest overall effects (Penner and Paul, 2017).

The pathomechanisms underlying pathological fatigue are only partially understood (Bower, 2014, Penner and Paul, 2017, Davies et al., 2021, Campos et al., 2022, Marshall-Gradisnik and Eaton-Fitch, 2022, Davis et al., 2023). A role of inflammatory processes, stress, and related disturbances in the hypothalamic–pituitary–adrenal axis has been discussed (Bower, 2014, Penner and Paul, 2017, Davies et al., 2021, Campos et al., 2022, Marshall-Gradisnik and Eaton-Fitch, 2022, Davis et al., 2023). In addition, (immune-mediated) dopaminergic and serotonergic signaling changes have been related to fatigue (Dantzer et al., 2014, Heitmann et al., 2022). How these mechanisms differentially contribute to fatigue in different disorders is unclear so far. However, since the symptomatology of fatigue is similar at the behavioral level, different mechanisms likely converge and translate into brain network dysfunction, ultimately resulting in neuropsychiatric symptoms, including fatigue (Dantzer et al., 2014, Penner and Paul, 2017, Heitmann et al., 2022, Scangos et al., 2023). Beyond, the term fatigue is also used in healthy persons undergoing mentally or physically strenuous tasks. However, these transient states and the underlying pathology likely differ from pathological fatigue as a symptom of the abovementioned disorders.

A better understanding of the underlying pathophysiology at the brain network level will aid the development of reliable and valid biomarkers to improve the diagnosis and treatment of fatigue (Davies et al., 2021). Biomarkers can serve different functions. For instance, they can support the diagnosis and monitoring of symptoms and the prediction of treatment outcomes (BEST, 2016). Diagnostic biomarkers of fatigue might be particularly valuable as patients often face claims of malingering (McInnis et al., 2014). Moreover, such biomarkers might help to define new treatment targets. Since fatigue occurs in different disorders, a common transdiagnostic biomarker would be particularly appealing (Davies et al., 2021).

Electroencephalography (EEG) is particularly promising for developing a transdiagnostic biomarker of fatigue since it is broadly available, non-invasive, cost-effective, and potentially mobile. Moreover, EEG-based biomarkers might serve as treatment targets for (non-invasive) neuromodulation techniques that have already shown first promising results in fatigue (Krawinkel et al., 2015, Lefaucheur et al., 2017). Next to EEG, magnetoencephalography (MEG) is a technique that measures brain signals and can also be used to investigate neural function in pathological fatigue. Yet, the potential of M/EEG biomarkers for fatigue has not been assessed systematically. One systematic review assessed abnormalities in patients suffering from CFS assessed by different neuroimaging modalities, including EEG. The review reported largely inconsistent results from 11 EEG studies in sleeping and awake patients, and no synopsis for the various EEG measures has been provided (Maksoud et al., 2020). Another systematic review and meta*-*analysis assessed EEG correlates of mental fatigue in healthy participants undergoing cognitively demanding tasks. The results indicated increased theta and alpha band activity, predominantly in frontal and central brain regions (Tran et al., 2020). However, how these findings in healthy participants relate to pathological fatigue in patients remains unclear.

We, therefore, performed a systematic review of M/EEG recordings during the resting state in awake patients suffering from fatigue due to different disorders. This systematic review is intended to provide insights into brain function in fatigue. Beyond it can advance the development of transdiagnostic biomarkers of fatigue to eventually improve the assessment and treatment of this frequent and burdensome symptom.

## 2 Methods

The present review was performed and reported according to the Preferred Reporting Items for Systematic Reviews and Meta-Analyses Guidelines (PRISMA) (Page et al., 2021). The protocol was preregistered on PROSPERO on the 3rd of May 2022 (CRD42022330113). Record deduplication, title and abstract screening, full-text review, and data extraction were performed using the software Covidence (Veritas Health Innovation 2021, Melbourne, Australia).

### 2.1 Search strategy

The databases MEDLINE (via PubMed), Web of Science Core Collection (via Web of Science), and EMBASE (via Ovid) were searched on the 3rd of May 2022 and again before the final analysis on the 9th of September 2022. No time limit was applied. For EMBASE and Web of Science Core Collection, the search was limited to Articles. Moreover, we screened reference lists of included studies for further relevant publications.

The search string used a combination of the term “fatigue” and related terms with “electroencephalography” or “magnetoencephalography” and associated terms. The entire search strategy can be found in the supplementary material.

### 2.2 Study selection

The inclusion and exclusion criteria for the study can be found in Table 1. In summary, peer-reviewed studies using quantitative resting-state M/EEG to analyze brain activity in relation to pathological fatigue were included.

**Table 1:** Inclusion and exclusion criteria.

**Inclusion (included, if all apply)**	**Exclusion (excluded, if any applies)**
Published, peer-reviewed study	Review article or case report
Humans >= 18 years old	Animal studies
Pathological fatigue as a symptom of disease or syndrome	Task-related fatigue/fatigability in healthy participants
Quantitative wake resting-state M/EEG	M/EEG during sleep

### 2.3 Record screening, full-text review, and data extraction

Two authors (H.H., V.D.H., P.T.Z., or M.P.) screened titles and abstracts, and performed the full-text review blinded to the other authors’ decision. A third author was consulted in case of disagreement, and conflicts were discussed. Data extraction was performed by one author (H.H.) and checked by another author (V.D.H. or P.T.Z.). Data extraction comprised study characteristics (sample size, sex, age, diagnostic entity, and clinical assessment tools), quantitative M/EEG measures (peak alpha frequency, frequency-specific power, frequency-specific connectivity), and study design according to Grimes and Schulz (Grimes and Schulz, 2002).

### 2.4 Data synthesis strategy

For data synthesis, studies were grouped concerning study design. A formal meta-analysis was not feasible due to the heterogeneity of reported outcome measures and study designs. Therefore, semiquantitative data synthesis was performed using modified albatross plots (Harrison et al., 2017). Albatross plots allow graphically estimating effect sizes for studies with similar research questions by plotting p-values against sample sizes for different directions of effects. Due to the heterogeneous statistical methods applied in the included studies, effect size estimation contours could not be superimposed on the plots. Modified albatross plots were used to present the results of cross-sectional studies comparing peak alpha frequency (PAF), frequency-specific power at delta, theta, alpha, beta, and gamma frequencies, as well as frequency-specific connectivity at theta, alpha, beta, and gamma frequencies in patients versus healthy participants. If a single study reported multiple p-values for different brain regions of interest, the lowest p-value was extracted. P-values were depicted on the x-axis and reported as in the primary studies, independently of possible multiple comparison adjustments. In case of imprecise p-value reporting (e.g., p < 0.05), modified albatross plots show the nearest decimal (e.g., p = 0.049). The modified albatross plots used in the present study (see Fig. 4, Fig. 5, Fig. 6) depict higher values for the variable of interest in patients compared to healthy participants on the right-hand side, non-significant results in the middle, and lower values on the left-hand side of each panel. The total sample size of each study was depicted on the y-axis. Narrative data synthesis was applied for cross-sectional studies reporting quantitative M/EEG measures other than the variables mentioned above (e.g., classification approaches, graph-theory-based measures, microstates) and descriptive and longitudinal studies. This was due to the low number of studies and the high heterogeneity of methods and outcome measures.

### 2.5 Risk of bias and quality assessment

Risk of bias and quality assessment of included studies was performed using a modified version of the Newcastle-Ottawa Scale (Lo et al., 2014) as previously reported (Zebhauser et al., 2022). Domains used to assess the risk of bias and quality of studies comprised “selection of study participants” (4 items), “comparability/confounders” (2 items), and “outcome data” (3 items). Items were rated as “high” risk of bias (negative for study quality) or “low” risk of bias (positive for study quality) to allow for a more straightforward interpretation of scoring results. Sum scores for single studies were not calculated because single items frequently had to be scored “n/a” (not applicable) and thus would have led to a misleading comparison of sum scores across studies. The assessment of the risk of bias and study quality was performed by one author (H.H.) and checked by another author (V.D.H. or P.T.Z.).

## 3 Results

### 3.1 Study selection

**Fig. 1:**
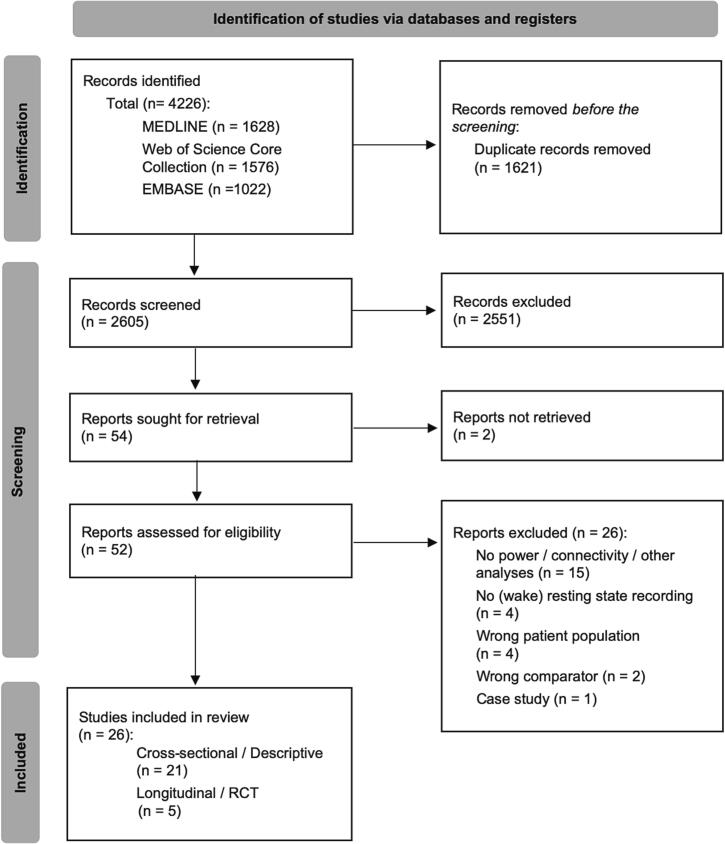
**PRISMA Flowchart of study selection.** PRISMA, preferred reporting items for systematic reviews and meta-analyses; RCT, randomized controlled trial.

Two thousand six hundred five records were identified after deduplication. Title and abstract screening identified 52 records for full-text review. After full-text review, 26 studies were included in the final analyses (Bruno et al., 1998, Kravitz et al., 2006, Sherlin et al., 2007, Flor-Henry et al., 2010, Kayıran et al., 2010, Duffy et al., 2011, Loganovsky, 2011, Neu et al., 2011, Moore et al., 2014, Cogliati Dezza et al., 2015, Navarro Lopez et al., 2015, Gschwind et al., 2016, Wu et al., 2016, Zinn et al., 2016, Buyukturkoglu et al., 2017, Vecchio et al., 2017, Zinn et al., 2017, Fallon et al., 2018, Jensen et al., 2018, Zinn et al., 2018, Golonka et al., 2019, Park et al., 2019, Porcaro et al., 2019, Sjøgård et al., 2021, Zinn and Jason, 2021, Zinn et al., 2021). For details on the study selection process and reasons for exclusion in the different stages, see the PRISMA flow diagram in Fig. 1.

### 3.2 Study characteristics

**Fig. 2:**
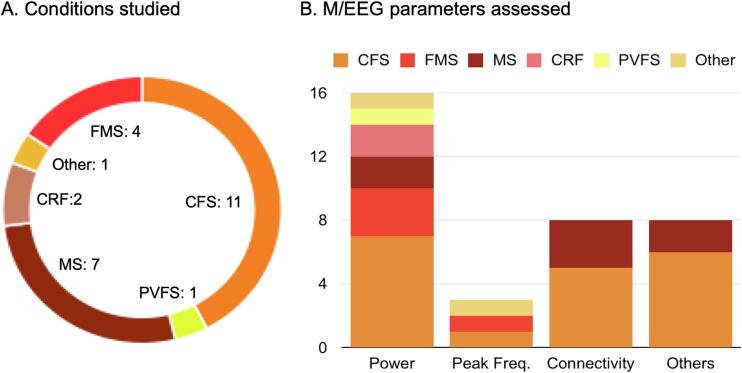
**Conditions and M/EEG parameters.** The figure shows counts for the conditions studied in records included (Panel A) and M/EEG parameters assessed (Panel B). CFS, chronic fatigue syndrome; CRF, cancer-related fatigue; FMS, fibromyalgia syndrome; MS, multiple sclerosis; PVFS, post-viral fatigue syndrome; M/EEG, magneto-/electroencephalography.

Study characteristics are depicted in Fig. 2 and summarized in Table 2. Twenty-one studies had a cross-sectional or descriptive study design, and five had a longitudinal or RCT design. Eleven studies were conducted in patients with Chronic Fatigue Syndrome (CFS), seven in patients with Multiple Sclerosis (MS), four studies in patients with Fibromyalgia Syndrome (FMS), two studies in patients with Cancer-related Fatigue (CRF), one study in patients with Post-viral Fatigue Syndrome (PVFS), and one study in other conditions (Burnout Syndrome). Twenty-five studies used EEG, whereas only one study used MEG. M/EEG power was assessed in 16 studies, peak frequency in three studies, connectivity in eight studies, and other measures, including graph-theory-based brain network properties and classification approaches in another eight studies. Sample sizes ranged from 13 to 460 (median 36). Studies were published between 1998 and 2021.

**Table 2:** Summary of study characteristics.

**Author and year**	**Study design**	**Inter-vention**	**M/EEG**	**Entity**	**Total sample size**	**Assess-ment tool**	**M/EEG Analysis**	**Main result (M/EEG)**
Bruno 1998 (Bruno et al., 1998)	Descriptive	–	EEG	PVFS	33	PFQ	Power	Power equal between hemispheres across all frequency bands EEG slow wave power in the right hemisphere significantly correlated with daily fatigue scores
Buyukturkoglu 2017 (Buyukturkoglu et al., 2017)	Cross-sectional observational	–	EEG	MS	29	mFIS	Connectivity	FC (Coherence) differing in various frequency bands and brain regions between fatigued MS patients and healthy participants Coherence in the theta and beta band in the fronto-frontal region as well as in the beta band in the temporo-parietal region positively correlated with fatigue scores
Cogliati Dezza 2015 (Cogliati Dezza et al., 2015)	Cross-sectinal observational	–	EEG	MS	35	mFIS	Power	Lower right hemisphere power in patients with high but not low fatigue compared to healthy participantsInter-hemispheric total power (L > R) of homologous sensorimotor (SM1) areas positively correlated with fatigue scores
Duffy 2011 (Duffy et al., 2011)	Cross-sectional observational	–	EEG	CFS	460	–	Other (Classification)	Highly significant group discrimination in unmedicated females (86.8% patients and 89.8% HC accuracy) and males (88.9% patients and 82.4% healthy participants accuracy)Less accurate discrimination in patients taking psychoactive medications (females 77.8%, males 60.0%) Bilateral temporal lobe involvement in 9/10 discrimination factors
Fallon 2018 (Fallon et al., 2018)	Cross-sectional observational	–	EEG	FMS	37	–	Power	Higher theta power in prefrontal and anterior cingulate cortices in patients compared to healthy participants Positive correlation of theta changes with tiredness, tenderness and pain scores
Flor-Henry 2010 (Flor-Henry et al., 2010)	Cross-sectional observational	–	EEG	CFS	137	–	Power/PF/Other (Classification)	Lower alpha power in the parieto-occipital region and lower beta power in the fronto-temporal region in patients compared to healthy participants No significant PF differences Classification approach using spectral current density in the alpha and beta band with 72% and 71% accuracy, respectively
Golonka 2019 (Golonka et al., 2019)	Cross-sectional observational	–	EEG	Other (Burnout)	95	–	Power/PF	Lower alpha power in patients compared to healthy participants Alpha power negatively correlated with exhaustion symptoms in anterior and posterior region No significant PF difference
Gschwind 2016 (Gschwind et al., 2016)	Cross-sectional observational	–	EEG	MS	102	FSMC	Other (Microstate)	Cognitive fatigue significantly predicted by short duration of class B microstate
Jensen 2018 (Jensen et al., 2018)	(Randomized) Controlled Trial	NFB + Hypnosis vs. MM+ Hypnosis vs. Hypnosis	EEG	MS	32	FSS	Power	Hypnosis increased theta, beta and gamma power only in patients who received NFB and decreased beta and gamma in those who received MM
Kayiran 2010 (Kayıran et al., 2010)	(Randomized) Controlled Trial	NFB vs. Escitalopram	EEG	FMS	36	VAS	Power	No power changes in NFB compared to control group Decrease in theta/sensory motor rhythm ratio in NFB group
Kravitz 2006 (Kravitz et al., 2006)	(Randomized) Controlled Trial	NFB vs. Sham	EEG	FMS	47	Fibro-myalgia Symptom Scales	Power	Pre-treatment delta/alpha amplitude ratio > 1 associated with participant rated but not clinician rated global impression response, independent of intervention
Loganovsky 2000 (Loganovsky, 2011)	Cross-sectional observational	–	EEG	CFS	38	–	Power	Lateralized (left-sided) in- crease of theta and beta as well as decrease of alpha power
Lopez 2015 (Navarro Lopez et al., 2015)	Cross-sectional observational	–	EEG	FMS	26	–	Power/PF	Ratios of theta/alpha and beta/alpha power as indicators of disease severity PF higher in patients compared to healthy participants
Moore 2014 (Moore et al., 2014)	Longitudinal descriptive	Chemo-therapy	EEG	CRF	18	BFI	Power	Total spectrum power increased after a physical task in patients during chemotherapy but not healthy participants
Neu 2011 (Neu et al., 2011)	Cross-sectional observational	–	EEG	CFS	30	FSS	Power	Higher theta power in electrodes Fp1 and F4 as well as higher beta power in electrode O2 in patients compared to healthy participants
Park 2019 (Park et al., 2019)	Descriptive	–	EEG	CRF/CFS	45	BFI/FSS	Power	FSS scores positively correlated with frontal delta, theta, alpha power in CFS group
Porcaro 2019 (Porcaro et al., 2019)	(Randomized) Controlled Trial	tDCS vs. Sham	EEG	MS	30	mFIS	Connectivity	Before treatment, more severely impaired resting state dynamics in S1 than in M1 in fatigued patients Left S1 fractal dimension at rest impaired compared to healthy participants before but not after tDCS treatment
Sherlin 2007 (Sherlin et al., 2007)	Cross-sectional observational	–	EEG	CFS	34	–	Power	Higher delta power in the left uncus and parahippocampal gyrus as well as higher theta power in the cingulate gyrus and right precentral gyrus of the frontal lobe in patients compared to HC twins
Sjøgård 2021 (Sjogard et al., 2021)	Cross-sectional observational	–	MEG	MS	146	FSMC	Connectivity	Lower alpha FC within the DMN and between the DMN, SMN and LAN as well as lower interhemispheric beta FC among nodes of the SMN in patients compared to healthy participants Significant negative correlation of FC with cognitive fatigue
Vecchio 2017 (Vecchio et al., 2017)	Cross-sectional observational	–	EEG	MS	38	mFIS	Other (Graph measures)	Fatigue symptoms positively correlated with beta smallworldness in SN
Wu 2016 (Wu et al., 2016)	Cross-sectional observational	–	EEG	CFS	47	–	Power	Delta, theta and alpha power increased in frontal and prefrontal brain regions of patients compared to healthy participants Overall decrease in intensity and complexity of the brain electrical signals in patients
Zinn 2016 (Zinn et al., 2016)	Cross-sectional observational	–	EEG	CFS	18	–	Power/Connectivity	Decreased alpha power in bilateral parietal, occipital and posterior temporal lobes in patients compared to healthy participants Significantly decreased lagged phase synchronization for delta and alpha including the DMN and CEN
Zinn 2017 (Zinn et al., 2017)	Cross-sectional observational	–	EEG	CFS	29	DSQ	Connectivity/Other (Graph measures)	Lower delta smallworldness in patients compared to healthy participants Delta smallworldness negatively correlated with neurocognitive impairment scores on the DSQ
Zinn 2018 (Zinn et al., 2018)	Cross-sectional observational	–	EEG	CFS	100	MFI-20/FSS	Power	Increased delta power predominately in the frontal lobe, and decreased beta power in the medial and superior parietal lobe in patients compared to healthy participants Left- lateralized, frontal delta sources associated with a clinical reduction in motivation
Zinn 2021a (Zinn et al., 2021)	Cross-sectional observational	–	EEG	CFS	13	DSQ	Power	Higher delta and lower alpha and beta power in patients compared to healthy participants
Zinn 2021b (Zinn and Jason, 2021)	Cross-sectional observational	–	EEG	CFS	68	DSQ	Other (Graph measures)	Significant group differences in baseline CAN organization Cognitive, affective, and somato-motor symptom cluster ratings associated with alteration to CAN topology in patients, depending on the frequency band

*Note.* M/EEG, Magneto-/Electroencephalography; PF, Peak Frequency; mFIS, modified Fatigue Impact Scale; FSS, Fatigue Severity Scale; FSMC, Fatigue Scale for Motor and Cognitive Funktions; DSQ, DePaul Symptom Questionnaire; BFI, Brief Fatigue Inventory; PFQ, Post-Polio Fatigue Questionnaire; NFB, Neurofeedback; MM, Mindfulness Meditation; tDCS, transcranial direct current stimulation; FC: Functional Connectivity; CEN, Central executive network; DMN: Default-mode network; SN, Salience Network; SMN; Sensory-motor network; CAN, Central autonomic network; LAN, language network; rsFC, resting-state functional connectivity; S1, primary somatosensory cortex; M1, primary motor cortex; CFS, chronic fatigue syndrome; CRF, cancer-related fatigue; FMS, fibromyalgia syndrome; MS, multiple sclerosis; PVFS, post-viral fatigue syndrome.

### 3.3 Risk of bias assessment

**Fig. 3:**
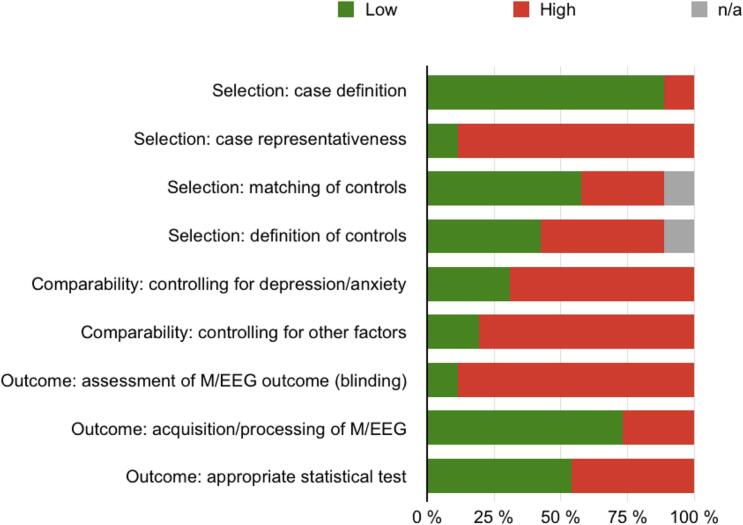
**Risk of bias assessment for included studies.** M/EEG, magneto-/electroencephalography.

Results from the risk of bias assessment are presented in Fig. 3. Individual study scores are shown in the supplementary Table S1. In the domain “selection of study participants,” case definitions were clearly specified in most studies. The most significant bias was “case representativeness” due to lacking a specific sampling/recruiting strategy. Additionally, the selection and definition of healthy participants were partially not clearly described. In the “comparability” domain, many studies assessed depression/anxiety and other closely related symptoms. However, the majority did not control for these parameters or formally include them in their analysis, leading to a particularly “high” risk of bias in this domain. In the “outcome” domain, the most significant risk of bias arose for the “assessment of M/EEG outcomes” due to manual data processing with insufficient information regarding blinding procedures, e.g., for condition and clinical data. Furthermore, nearly half of the studies did not describe the statistical tests in detail and did not include statements on corrections for multiple comparisons.

### 3.4 Data synthesis

Modified albatross plots were used for semiquantitative data analysis from cross-sectional studies reporting power, connectivity, or peak frequency results. Narrative data synthesis was used for all other cross-sectional studies using less common analysis techniques and outcome measures (e.g., graph-theory-based measures, classification approaches, and microstate analysis) as well as descriptive and longitudinal studies.

#### 3.4.1 Semiquantitative data analysis of cross-sectional studies reporting power, peak frequency, or connectivity results

Eleven cross-sectional studies with a sample size between 13 and 137 (median 37) assessed power; of those 11, three studies (sample size 26–137) also reported peak frequencies. Three additional studies (sample size 18–146) assessed connectivity measures.

##### 3.4.1.1 Power

**Fig. 4:**
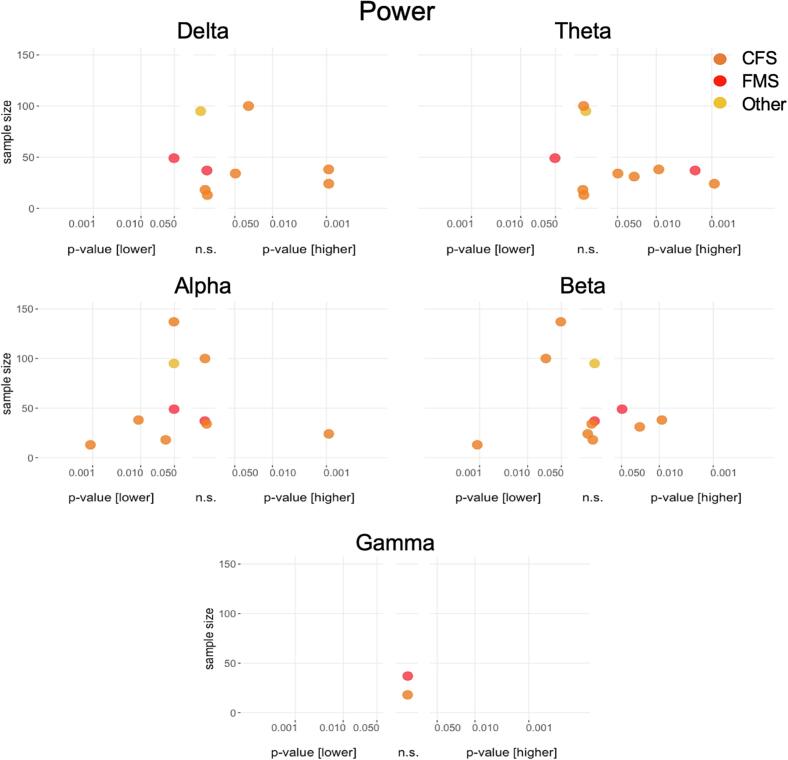
**Results of cross-sectional analyses of power for the different frequency bands.** Power differences between patients and healthy participants in cross-sectional studies. P values on the x-axis are displayed on a logarithmic scale (log10). Higher values in patients compared to healthy participants are depicted on the right-hand side, non-significant differences in the middle and lower values on the left-hand side of each panel. The total sample size for single studies is depicted on the y-axis. n.s., not significant.

Power results are shown in Fig. 4. Delta power was assessed in nine studies. Four studies (all in patients with CFS), depicted on the right-hand side of the panel for delta power, showed higher power in patients compared to healthy participants (Sherlin et al., 2007, Loganovsky, 2011, Wu et al., 2016, Zinn et al., 2018). Four studies, depicted in the middle of the delta power panel, yielded non-significant results (Zinn et al., 2016, Fallon et al., 2018, Golonka et al., 2019, Zinn et al., 2021). One study in patients with FMS, depicted on the left-hand side of the panel, reported lower delta power (Navarro Lopez et al., 2015). Theta power was tested in ten studies. Five studies (four CFS, one FMS) reported higher power in patients compared to healthy participants (Sherlin et al., 2007, Loganovsky, 2011, Neu et al., 2011, Wu et al., 2016, Fallon et al., 2018); four studies showed non-significant results (Zinn et al., 2016, Zinn et al., 2018, Golonka et al., 2019, Zinn et al., 2021) and one study in patients with FMS reported lower power (Navarro Lopez et al., 2015). Alpha power was assessed in ten studies. Six studies reported lower power in patients compared to healthy participants (four CFS, one FMS, one Other) (Flor-Henry et al., 2010, Loganovsky, 2011, Navarro Lopez et al., 2015, Zinn et al., 2016, Golonka et al., 2019, Zinn et al., 2021), three studies showed non-significant results (Sherlin et al., 2007, Fallon et al., 2018, Zinn et al., 2018) one study in patients with CFS showed higher power (Wu et al., 2016). Beta power was tested in all eleven studies, with three studies (all in patients with CFS) reporting lower power (Flor-Henry et al., 2010, Zinn et al., 2018, Zinn et al., 2021), and five studies (three CFS, one FMS, and one Other) with non-significant results (Sherlin et al., 2007, Wu et al., 2016, Zinn et al., 2016, Fallon et al., 2018, Golonka et al., 2019) and three studies (two FCS, one FMS) showing higher beta power values (Loganovsky, 2011, Neu et al., 2011, Navarro Lopez et al., 2015). Gamma power was assessed in two studies (one CFS, one FMS), with both studies reporting non-significant results (Zinn et al., 2016, Fallon et al., 2018).

**Fig. 5:**
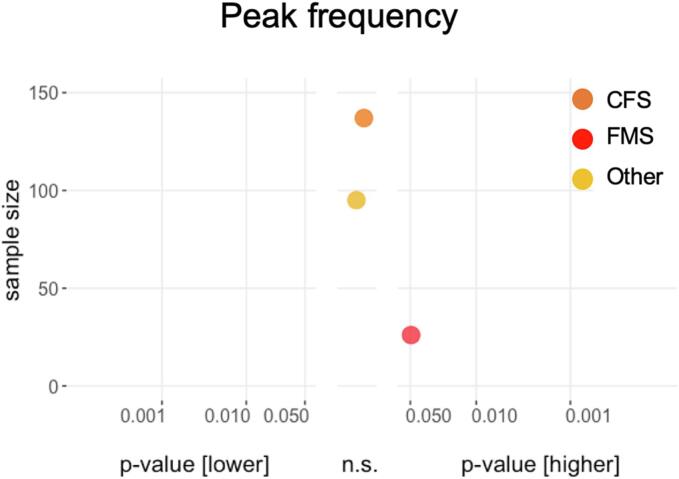
**Results of cross-sectional peak frequency analyses.** Differences between patients and healthy participants in cross-sectional studies. P values on the x-axis are displayed on a logarithmic scale (log10). Higher values in patients compared to healthy participants are depicted on the right-hand side, non-significant differences in the middle and lower values on the left-hand side. The total sample size for single studies is depicted on the y-axis. n.s., not significant.

**Fig. 6:**
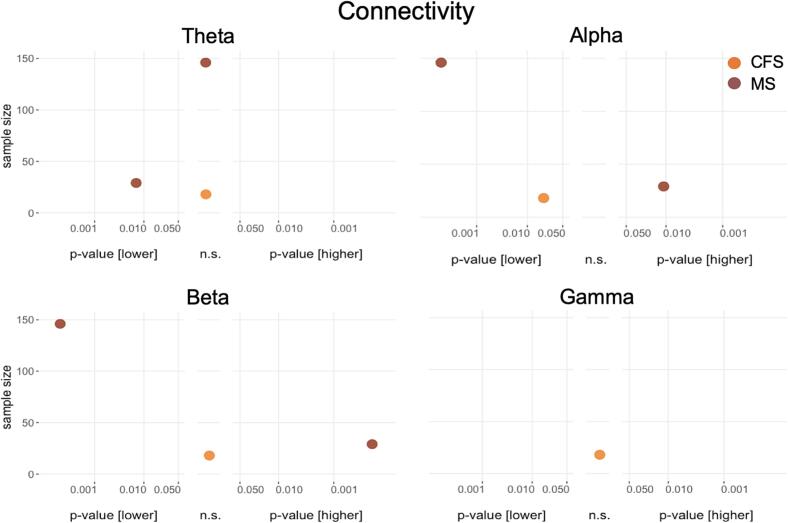
**Results of cross-sectional connectivity analyses for the different frequency bands.** Differences between patients and healthy participants in cross-sectional studies. P values on the x-axis are displayed on a logarithmic scale (log10). Higher values in patients compared to healthy participants are depicted on the right-hand side, non-significant differences in the middle and lower values on the left-hand side of each panel. The total sample size for single studies is depicted on the y-axis. n.s., not significant.

In summary, more studies reported higher theta power and lower alpha power in patients compared to healthy participants. Results were inconclusive for delta, beta, and gamma power.

##### 3.4.1.2 Peak frequency

Peak frequency results are shown in Fig. 5. One study reported a higher peak frequency in patients with FMS compared to healthy participants (Navarro Lopez et al., 2015). The other two studies (CFS and Other) showed non-significant results (Flor-Henry et al., 2010, Golonka et al., 2019). Thus, results for peak frequency were sparse and inconclusive.

##### 3.4.1.3 Connectivity

Connectivity results are shown in Fig. 6. Theta connectivity was assessed in three studies, with one study in patients with MS showing lower connectivity in patients compared to healthy participants (Buyukturkoglu et al., 2017) and the two other studies (one MS, one CFS) reporting non-significant results (Zinn et al., 2016, Sjøgård et al., 2021). Connectivity in the alpha band was reported in three studies, with two studies (one MS, one CFS) showing lower (Zinn et al., 2016, Sjøgård et al., 2021) and the other one (MS) showing higher alpha connectivity (Buyukturkoglu et al., 2017). Beta connectivity was assessed in three studies. One study in patients with MS showed lower (Sjogard et al., 2021) and one higher beta connectivity (Buyukturkoglu et al., 2017), and the other study in patients with CFS (Zinn et al., 2016) non-significant results in patients compared to healthy participants. Only one study in patients with CFS assessed gamma connectivity, yielding non-significant results (Zinn et al., 2016). Taken together, results for connectivity were sparse and inconclusive.

#### 3.4.2 Narrative synthesis of data analyses from cross-sectional studies using other outcome parameters as well as descriptive and longitudinal studies

##### 3.4.2.1 Cross-sectional studies using network analysis, microstate analyses, and classification approaches

Three studies (one MS, two CFS) assessed graph-theory-based network measures (Vecchio et al., 2017, Zinn et al., 2017, Zinn and Jason, 2021). Two studies evaluated the measure of smallworldness in patients with CFS and MS. In patients with MS, a positive correlation of smallworldness in the beta band with fatigue symptoms was found (Vecchio et al., 2017). In patients with CFS, smallworldness in the delta band was reduced compared to healthy participants, which also negatively correlated with neurocognitive impairment (Zinn et al., 2017). A third study found a significant group difference in the topology of the cortico-autonomic network (CAN) in patients with CFS compared to healthy participants, which further showed associations with cognitive, affective, and somatomotor symptoms in different frequency bands (Zinn and Jason, 2021).

Two studies reported machine-learning approaches based on power measures for classification in patients with CFS (Flor-Henry et al., 2010, Duffy et al., 2011). One study reported significant differences between medicated and unmedicated as well as female and male patients, with accuracies ranging from 60 to 89 % for patients with and without psychoactive medication, respectively (Duffy et al., 2011). Another study reported accuracies of 72 and 71 % using spectral current density in the alpha and beta bands, respectively (Flor-Henry et al., 2010).

One microstate analysis study found that a short duration of microstate B significantly predicted cognitive fatigue in patients with MS.

Together, these studies suggest fatigue-related alterations on the network level at different frequencies. However, the findings were too heterogenous to infer specific EEG network abnormalities in fatigue.

##### 3.4.2.2 Descriptive studies using power analysis

Four descriptive studies reported the relationship of EEG power with fatigue symptoms. Three studies (one PVFS, MS, and CFS) reported cross-sectional relationships, yielding inconsistent results. In one study in patients with PVFS, EEG slow-wave power in the right hemisphere significantly correlated with daily fatigue severity (Bruno et al., 1998). In one study in patients with MS, fatigue positively correlated with the inter-hemispheric power asymmetry of primary sensorimotor cortices as assessed with the inter-hemispheric symmetry index (Cogliati Dezza et al., 2015). In patients with CFS, frontal delta, theta, and alpha power were found to correlate with fatigue scores positively (Park et al., 2019). One longitudinal descriptive study in patients with CRF did not report changes in resting-state EEG power (Moore et al., 2014). Together, these findings were too heterogenous to draw conclusions about EEG correlates of fatigue.

##### 3.4.2.3 (Randomized) controlled trials using neurofeedback and non-invasive brain stimulation (NIBS)

Four (randomized) controlled trials investigated the impact of neuromodulatory interventions on EEG measures and/or fatigue symptoms. Three studies (one MS and two FMS) reported power changes after neurofeedback (NFB) interventions with different control conditions and inconsistent results (Kravitz et al., 2006, Kayıran et al., 2010, Jensen et al., 2018).

One study assessed the effects of transcranial direct current stimulation (tDCS) on connectivity in patients with MS suffering from fatigue. This study reported normalization of impaired resting-state fractal dimension, which encodes network complexity, of primary sensorimotor brain areas compared to healthy participants after the intervention (Porcaro et al., 2019).

Taken together, studies assessing EEG correlates of neurofeedback interventions and NIBS for fatigue were too heterogenous to provide compelling evidence.

## 4 Discussion

### 4.1 Main findings

The present systematic review assessed potential transdiagnostic M/EEG biomarkers of pathological fatigue. To this end, cross-sectional as well as descriptive and longitudinal studies reporting resting-state M/EEG findings in awake patients suffering from fatigue were analyzed. The main finding from cross-sectional studies, which can serve to identify diagnostic biomarkers, was an increase in theta power and a decrease in alpha power. The results of descriptive and longitudinal studies were too heterogenous to draw firm conclusions about M/EEG abnormalities in fatigue.

A previous systematic literature review assessed neuroimaging alterations from different modalities, including EEG, in patients suffering from CFS (Maksoud et al., 2020). For 11 EEG studies in sleeping and awake patients, the review reported largely inconsistent results for different EEG measures, including power and functional connectivity. The review did not present a synopsis or (semi-)quantitative analysis but discussed findings from selected studies, which were interpreted to suggest an overall decreased EEG activity in patients suffering from CFS (Maksoud et al., 2020). Moreover, functional connectivity changes in the delta, theta, and alpha band were discussed as a potential sign of decreased complexity and an inhibitory state of brain function (Wu et al., 2016, Maksoud et al., 2020). Another systematic review and meta-analysis assessed EEG spectral power as a potential biomarker of task-related mental fatigue in healthy participants (Tran et al., 2020). In the 21 studies included, the authors found a robust correlation between mental fatigue with increases in theta and alpha band activity, with large and moderate to large effect sizes, respectively. This effect was most pronounced in the frontal, central, and posterior brain regions. Functionally, this increase was interpreted as an inhibitory mechanism with reference to studies on memory and cognition (Sauseng et al., 2010, Tran et al., 2020).

The present systematic review complements and extends these findings by suggesting a shift towards brain activity at lower frequencies in patients suffering from fatigue across different disorders. Theta oscillations have been implicated in alertness and cognitive control (Klimesch, 1999, Sauseng et al., 2010, Snipes et al., 2022), and a reciprocal relationship of theta and alpha oscillations has been observed (Klimesch, 1999). In particular, higher theta and lower alpha activity during the resting state were associated with poorer cognitive performance (Klimesch, 1999), corresponding to the clinical presentation of fatigue (Penner and Paul, 2017). Additionally, (mid-frontal) theta oscillations have been implicated in signaling predictions and response conflicts (Cohen and Donner, 2013, Huang et al., 2015). This fits well with predictive coding approaches to fatigue, proposing that this symptom results from a perceived inability to counteract conflicting expectations and sensory inputs (Stephan et al., 2016, Henningsen et al., 2018).

The increase in slow-wave oscillations in fatigue in the present study overlaps with M/EEG findings in the highly comorbid symptoms of pain and depression. A recent systematic review reported increased theta power in chronic pain patients compared to healthy participants (Zebhauser et al., 2022). Another review reported robust delta and theta band power increases in patients suffering from depression and other neuropsychiatric disorders (Newson and Thiagarajan, 2018). This suggests partially overlapping brain oscillatory patterns in different neuropsychiatric disorders. A well-established concept to explain increased slow-wave oscillations in patients suffering from various neuropsychiatric disorders, including pain and depression, is the thalamocortical dysrhythmia model (Llinás et al., 1999, Llinás et al., 2005). This model proposes that abnormal thalamocortical theta oscillations cause subsequent alterations in higher frequency bands in the beta and gamma range, ultimately fostering different neuropsychiatric symptoms. In principle, the present findings in fatigue are compatible with this model.

### 4.2 Risk of bias and limitations

The interpretation of the present results is limited by a high risk of bias in many studies included. This refers to the low representativeness of cases, as reflected by relatively small sample sizes (median 36), with a priori sample size calculations being reported only rarely. Moreover, in many studies, methods and statistical tests applied to generate the outcomes were partially opaque, and largely unstandardized M/EEG (pre-)processing was used. Additionally, only a few studies effectively controlled for potential confounds such as neuropsychiatric comorbidities, medication effects, or demographic factors.

Further limitations apply to this systematic review itself. First, fatigue occurs in different disorders with different pathomechanisms. It is unclear whether and how these different pathomechanisms converge and result in similar behavioral symptomatology. Our finding of increased slow-wave brain activity might represent a neural level where these different pathomechanisms converge. Second, the present review aimed to include patient groups with different disorders. However, most studies included patients with CFS and MS, limiting the results' transdiagnostic generalizability. Correspondingly, the main findings obtained for power differences are driven by results in CFS patients. Still, the present review provides a comprehensive overview and synopsis of available data and methods and can thus serve as a basis for future studies on transdiagnostic biomarkers. This is particularly relevant given the rapidly increasing number of patients with long COVID. Third, the albatross plots used for semiquantitative data synthesis in the present study comprise local and global M/EEG measures. Thus, the contributions of specific brain regions and networks might be obscured. Fourth, the review is limited to semiquantitative evidence since formal meta-analysis was not feasible due to the overall low number of studies and the heterogeneous study designs and outcomes.

### 4.3 Outlook and recommendations

The present systematic review can aid the development of transdiagnostic biomarkers for pathological fatigue by guiding future M/EEG studies.

First, future studies should aim to assess biomarker specificity (Woo et al., 2017). Most previous studies did not adequately control for frequent comorbidities, including pain and depression. Thus, the specificity of the findings remains to be determined. Future studies should, therefore, formally assess and control for these symptoms. This might help to determine the specificity of the results and identify common pathophysiological mechanisms of comorbid neuropsychiatric symptoms in line with the National Institute of Mental Health (NIMH) research domain criteria (Insel et al., 2010). Specificity and sensitivity might be further enhanced by exploring composite biomarkers considering different biopsychosocial determinants of fatigue (Tracey et al., 2019). Exploring the composite value of inflammatory and neuroimaging biomarkers might be particularly promising in fatigue. Such an approach holds the potential to understand better overlapping (neuro-immune) pathomechanisms underlying the frequent comorbidity of fatigue, depression, and pain (Heitmann et al., 2022).

Second, a better understanding of M/EEG correlates of fatigue could help to define treatment targets for non-invasive brain stimulation (NIBS) techniques. This would complement recent evidence for the efficacy of frequency-specific NIBS in treating fatigue (Lefaucheur et al., 2017). NIBS showed promising results in MS and FMS fatigue (Lefaucheur et al., 2017, Ayache et al., 2022, Chen et al., 2022) and was recently proposed as a treatment for long COVID (Linnhoff et al., 2022).

Third, small sample sizes and opaque methodology hinder reproducibility, as in many of the studies included. Future studies should aim to enhance reproducibility in biomarker development by using large datasets and transparent and standardized data analysis (Munafo et al., 2017). In M/EEG research and beyond, this can be achieved by adhering to open science practices, including preregistration, data and code sharing to allow for multisite data analyses, the use of standardized reporting and data structuring (e.g., EEG-BIDS) (Pernet et al., 2019) as well as automated (pre-)processing and analysis pipelines (Pernet et al., 2020).

## 5 Conclusion

Insights into brain function in pathological fatigue promise to advance the understanding of the underlying pathophysiology and the development of biomarkers that could further the diagnosis and treatment of fatigue (Woo et al., 2017). Due to the broad availability and scalability, M/EEG holds excellent potential for developing such biomarkers. We, therefore, systematically reviewed the current evidence for such biomarkers. Cross-sectional studies yielded evidence that a shift towards lower frequency oscillations in the theta band might be helpful as a diagnostic biomarker of fatigue and might also represent a potential treatment target. Large-scale studies assessing different biomarker types and their specificity are needed to better exploit the potential of M/EEG as transdiagnostic biomarkers of fatigue. Adhering to the highest standards in transparency and reproducibility will be key to yield reliable results. Moreover, exploring conceptual and pathophysiological overlaps with comorbid depression and pain and developing composite biomarkers appears promising and might open new alleys for treatment.

## Declaration of Competing Interest

The authors declare that they have no known competing financial interests or personal relationships that could have appeared to influence the work reported in this paper.

## Data Availability

No data was used for the research described in the article.
