# Supplementary material for: Resting-state EEG and MEG biomarkers of pathological fatigue – A transdiagnostic systematic review
Source: Neuroimage Clin. 2023 Aug 18;39:103500. doi: 10.1016/j.nicl.2023.103500 (PMC10474495; doi:10.1016/j.nicl.2023.103500)
Supplement: Supplementary data 1 [file mmc1.docx]

**Supplementary Material – Resting-state M/EEG biomarkers of pathological fatigue**

**Search Strategy:**

PubMed:

("fatigue"[Title/Abstract] OR "fatigue"[MeSH Terms] OR "fatigue syndrome, chronic"[MeSH Terms]) AND ("EEG"[Title/Abstract] OR "electroencephalogra*"[Title/Abstract] OR "MEG"[Title/Abstract] OR "magnetoencephalogra*"[Title/Abstract] OR "electroencephalography"[MeSH Terms] OR "magnetoencephalography"[MeSH Terms])

Web of Science:

("fatigue") AND ("EEG" OR "electroencephalogra*" OR "MEG" OR "magnetoencephalogra*")

EMBASE:

(fatigue.ti. OR fatigue.ab.) AND (EEG.ti. OR EEG.ab. OR electroencephalogra$.ti. OR electroencephalogra$.ab. OR MEG.ti. OR MEG.ab. OR magnetoencephalogra$.ti. OR magnetoencephalogra$.ab.)

**Supplementary Table S1.** Individual study scores from the risk of bias assessment.

|  | **Selection of study participants** | | | | **Comparability / Confounders** | | **Outcome data** | | |
| --- | --- | --- | --- | --- | --- | --- | --- | --- | --- |
|  | **case definition** | **case representativeness** | **matching of controls** | **definition of controls** | **controlling for depression / anxiety** | **controlling for any other factor** | **assessment of outcome (M/EEG) - blinding** | **acquisition / processing (MEEG)** | **Appropriate statistical test** |
| **Bruno 1998** | Low | High | n/a | n/a | High | High | High | Low | Low |
| **Buyukturkoglu 2017** | Low | High | Low | High | Low | High | High | Low | Low |
| **Cogliati Dezza 2015** | Low | High | High | High | Low | High | High | High | High |
| **Duffy 2011** | Low | Low | Low | High | Low | Low | High | Low | Low |
| **Fallon 2018** | Low | High | Low | Low | High | High | High | Low | Low |
| **Flor-Henry 2010** | Low | High | High | Low | Low | Low | High | Low | High |
| **Golonka 2019** | High | High | Low | Low | High | High | High | Low | Low |
| **Gschwind 2016** | Low | High | Low | Low | Low | Low | High | Low | Low |
| **Jensen 2018** | Low | High | n/a | n/a | High | High | High | High | Low |
| **Kayiran 2010** | Low | High | n/a | n/a | High | High | High | Low | High |
| **Kravitz 2006** | Low | Low | Low | Low | High | High | Low | High | High |
| **Loganovsky 2000** | High | High | High | High | High | High | High | High | High |
| **Lopez 2015** | Low | High | Low | High | High | High | High | Low | High |
| **Moore 2014** | High | High | Low | Low | Low | High | High | High | High |
| **Neu 2011** | Low | High | Low | Low | High | High | High | High | High |
| **Park 2019** | Low | High | Low | Low | High | Low | High | Low | High |
| **Porcaro 2019** | Low | High | High | High | High | High | Low | Low | High |
| **Sherlin 2007** | Low | Low | Low | Low | High | High | Low | Low | High |
| **Sjøgård 2021** | Low | High | Low | Low | High | Low | High | Low | Low |
| **Vecchio 2017** | Low | High | Low | High | Low | High | High | Low | Low |
| **Wu 2016** | Low | High | Low | High | Low | High | High | High | High |
| **Zinn 2016** | Low | High | High | High | High | High | High | Low | Low |
| **Zinn 2017** | Low | High | High | High | High | High | High | Low | Low |
| **Zinn 2018** | Low | High | Low | Low | High | High | High | Low | Low |
| **Zinn 2021a** | Low | High | High | High | High | High | High | Low | Low |
| **Zinn 2021b** | Low | High | High | High | High | High | High | Low | Low |
